# A Deadly Kiss

**DOI:** 10.3201/eid2905.AC2905

**Published:** 2023-05

**Authors:** Byron Breedlove

**Affiliations:** Centers for Disease Control and Prevention, Atlanta, Georgia, USA

**Keywords:** art science connection, emerging infectious diseases, about the cover, public health, bacteria, diphtheria, Corynebacterium diphtheriae, Princess Alice, Princess Alice (1843−78), later Grand Duchess of Hesse, 1861, Franz Xaver Winterhalter, A Deadly Kiss, bacterial infections, vaccines, antibiotics, antitoxin, cutaneous diphtheria, respiratory infections

**Figure:**
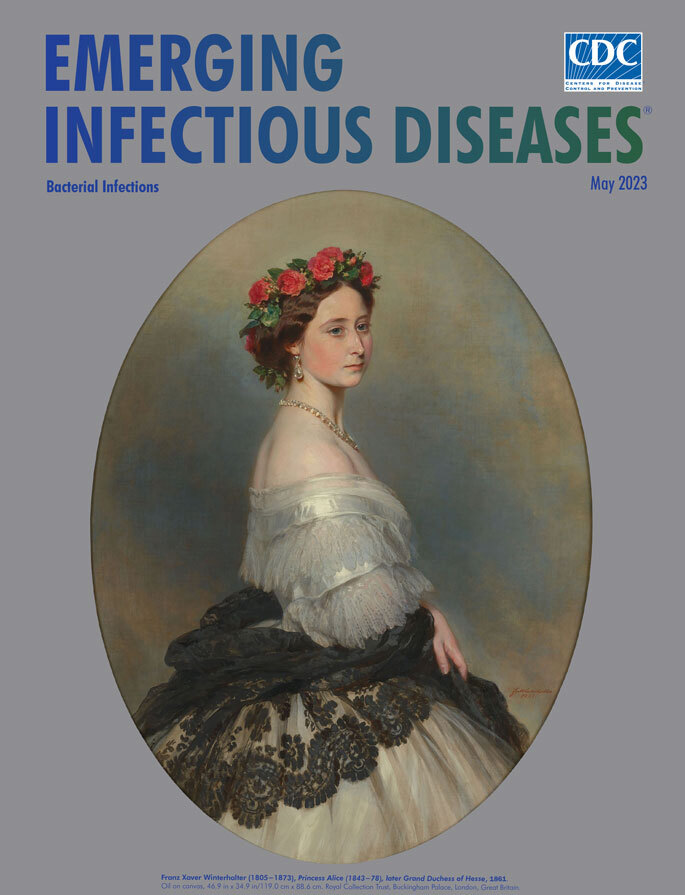
**Franz Xaver Winterhalter (1805−1873), *Princess Alice (1843−78), later Grand Duchess of Hesse*, 1861.** Oil on canvas, 46.9 in x 34.9 in/119.0 cm x 88.6 cm. Royal Collection Trust, Buckingham Palace, London, United Kingdom.

Diphtheria, a serious infection caused by the bacterium *Corynebacterium diphtheriae*, was described by Hippocrates early in the 5th century bce. In 1826, French physician Pierre Bretonneau named the disease *diphtérite*, derived from the Greek word for “leather” or “hide” because of the coating that appears in the throats of infected people. Before then, various names were ascribed to the disease or its aftermath, such as *El Año de los Garrotillos* (The Year of Strangulations) to characterize a 1613 epidemic in Spain. Before the availability of vaccines, diphtheria was a leading cause of childhood death. The most common type of diphtheria is the classic respiratory disease, and another type of infection, called cutaneous diphtheria, causes skin ulcers.

An outbreak of diphtheria in 1878 claimed many lives, not just among the youngest and poorest. Historically, diphtheria mortality rates have been typically higher among people in younger than older age groups. Among the victims were Princess Alice (Alice Maud Mary), whose 1861 portrait appears as this month’s cover image, and her young daughter, Marie. This painting is one of an estimated 120 portraits completed by German artist Franz Xaver Winterhalter for members of the English royal family. His exclusive clientele, which also included members of the royal families in Germany, France, and Belgium, made him a wealthy celebrity.

Born in Menzenschwand village in Germany’s Black Forest in 1805, Winterhalter was encouraged to draw from a young age. In 1818, he received art instruction in Fribourg, and in 1823, he moved to Munich to attend the Academy of Fine Arts, where one of his instructors was portraitist Josef Stieler. After traveling in Germany and Italy, Winterhalter moved to Paris and soon enjoyed the patronage of King Louis-Philippe. According to the Getty Museum, “In 1835, after he painted the German Grand Duke and Duchess of Baden, Winterhalter’s international career as a court portrait painter was launched.”

The Getty Museum notes that “Winterhalter’s portraits were prized for their subtle intimacy, but his popularity among patrons came from his ability to create the image his sitters wished or needed to project to their subjects. He was able to capture the moral and political climate of each court, adapting his style to each client until it seemed as if his paintings acted as press releases, issued by a master of public relations.”

Completed in June 1861, Winterhalter’s portrait shows a calm, beatific Princess Alice during an eventful year. Alice had acted as companion and caretaker for her maternal grandmother, Victoria, Duchess of Kent, who died in March. Alice’s engagement in April 1861 to Prince Louis IV of Hesse no doubt helped dispel the gloom from that loss. In December, however, Alice again found herself as caregiver, this time for her father, Prince Albert, who died in December. 

The Royal Trust Collection, home to this painting, notes that Alice “is wearing the dress in which she appeared at her first Drawing-Room (a formal reception where ladies were presented at court) on 7 May 1859. She was the third child and second daughter of Queen Victoria and Prince Albert. Known for her sweet nature, she often took on the role of peacekeeper in the royal household.” Alice, poised and composed, is wearing a white iridescent ball dress and a black shawl with a ringlet of flowers. Winterhalter’s skills are revealed by the textures and tones in Alice’s skin, hair, and clothes, the facets and reflections from her jewelry, and careful rendering of the flowers. 

After their wedding in 1862, Alice and Prince Louis moved to Germany. During the Austro-Prussian War in 1866, also known as Seven Weeks’ War, while Alice was pregnant with her third child, her father-in-law, Louis III, Grand Duke of Hesse, sided with the Austrians, and Prince Louis served as a commander for the Hessian Calvary, leaving Alice with the children. The Royal Museums Greenwich notes that “During this time, she befriended Florence Nightingale and played an active role in the region’s military hospitals.“ Afterward, Alice helped create a center to train nurses and sometimes personally helped care for the destitute. 

Prince Louis’ father and older brother both died in 1877, and Louis became Grand Duke and Alice Duchess of Hesse, a position she would only briefly hold. In November 1878, diphtheria spread throughout the royal household. The first stricken was Alice’s oldest daughter, Victoria, followed by Alix, Marie, Irene, Ernest, and her husband Louis. Marie died from diphtheria on November 15, although Alice initially did not reveal Marie’s death to her other children. When Ernest heard the news, he proved devastated; accounts suggest that Alice comforted her son with a kiss that would prove deadly. Whether that contact infected her remains a point of conjecture, but Alice nonetheless contracted diphtheria and died on December 14, 1878, the 17th anniversary of her father’s death. 

Five years after Alice’s death, bacteriologists Edwin Klebs and Friedrich Löffler identified the bacterium that causes diphtheria and named it Klebs–Löffler bacillus (now called *Corynebacterium diphtheriae*). Diphtheria is now largely preventable by an effective vaccine regimen and treatable with antibiotics and an antitoxin. Recent data from the World Health Organization reported that, in 2021, there were 8,638 cases globally. Although diphtheria is now rare in industrialized nations and its global burden has declined dramatically, in locales where vaccination coverage is inadequate or vaccination is disrupted or unavailable because of conflict, population displacement, or misinformation, recent outbreaks have been reported and resurgence of diphtheria remains possible.
